# 

**Published:** 1887-01-01

**Authors:** 


### CONTENTS.

**The New Year: The Association and "The Hospital"** - - - - - - 225

**Words of Consolation.**—XIV. Sympathy - - 226

**A Noble Profession: Nursing the Sick.**—The System of Nursing at the London Hospital. By Miss EVA C. E. LUCKES, Matron - - - - 227

**Sister Olive: a Hospital Romance.**—By W. J. ECCOTT. *Concluded* - - - - - - - 229

**Remedies: New and Old** - - - - 231

**Notes and News.**—Miscellaneous.—Donations.—Fancy Dress Ball in Aid of the Chelsea Hospital for Women.—The City Orthopædic Hospital.—Guy's Hospital.—Entertainment in Aid of Charing Cross Hospital.—New Home for English and American Girls in Berlin.—Ancient Order of Foresters' Convalescent Home. — Queen Victoria Maternity Hospital at Bristol.—Hospital Appeals.—The Great Northern Central Hospital.—Competition among Doctors. — The Ancoats Hospital. — The Children's Ministering Angels League.—The Hull Children's Hospital.—Testimonial to Sir Andrew Clark.—The Thronemount Consumption Hospital, etc., etc. - - - 232

**Should "Sisters" and "Nurses" Marry?** The Story of "Sister Olive" - - - - - 236

**The Gratitude of Hospital Patients.** By a Practical Philanthropist - - - - - 237

**Till the Doctor Comes.**—VII. Feeding.—Preservation of Ice - - - - - - - 238

**Flowers, Ferns, and Ward Decorations** - 239

**In- and Out-Patients' Hospital Diary** - - 240

**The Children's Corner.**—Puzzles, Answers, etc. - vi

**The Hospital Charity Box.**—Prizes and Results - vi

**Notices to Correspondents** - - - - vi

